# IRProfiler – a software toolbox for high throughput immune receptor profiling

**DOI:** 10.1186/s12859-018-2144-z

**Published:** 2018-04-18

**Authors:** Christos Maramis, Athanasios Gkoufas, Anna Vardi, Evangelia Stalika, Kostas Stamatopoulos, Anastasia Hatzidimitriou, Nicos Maglaveras, Ioanna Chouvarda

**Affiliations:** 1 Lab of Computing, Medical Informatics & Biomedical-Imaging Technologies, Department of Medicine, Aristotle University of Thessaloniki, 54124 Thessaloniki, Greece; 2 Institute of Applied Biosiences, Centre for Research & Technology Hellas, 57001 Thermi, Greece

**Keywords:** Immune receptor profiling, Software pipeline, High-throughput sequencing, B-cell receptors, T-cell receptors

## Abstract

**Background:**

The study of the huge diversity of immune receptors, often referred to as immune repertoire profiling, is a prerequisite for diagnosis, prognostication and monitoring of hematological disorders. In the era of high-throughput sequencing (HTS), the abundance of immunogenetic data has revealed unprecedented opportunities for the thorough profiling of T-cell receptors (TR) and B-cell receptors (BcR). However, the volume of the data to be analyzed mandates for efficient and ease-to-use immune repertoire profiling software applications.

**Results:**

This work introduces Immune Repertoire Profiler (IRProfiler), a novel software pipeline that delivers a number of core receptor repertoire quantification and comparison functionalities on high-throughput TR and BcR sequencing data. Adopting 5 alternative clonotype definitions, IRProfiler implements a series of algorithms for 1) *data filtering*, 2) calculation of *clonotype diversity and expression*, 3) calculation of *gene usage* for the V and J subgroups, 4) detection of *shared and exclusive clonotypes* among multiple repertoires, and 5) *comparison of gene usage* for V and J subgroups among multiple repertoires. IRProfiler has been implemented as a toolbox of the Galaxy bioinformatics platform, comprising 6 tools. Theoretical and experimental evaluation has shown that the tools of IRProfiler are able to scale well with respect to the size of input dataset(s). IRProfiler has been utilized by a number of recently published studies concerning hematological disorders.

**Conclusion:**

IRProfiler is made freely available via 3 distribution channels, including the Galaxy Tool Shed. Despite being a new entry in a crowded ecosystem of immune repertoire profiling software, IRProfiler founds its added value on its support for alternative clonotype definitions in conjunction with a combination of properties stemming from its user-centric design, namely ease-of-use, ease-of-access, exploitability of the output data, and analysis flexibility.

## Background

The huge diversity of antigen-specific receptors, most importantly the T-cell receptors (TR) on T cells and B-cell receptors (BcR) on B cells, endows the host with the ability to combat a wide range of pathogens. V(D)J recombination, i.e., the rearrangement of germline V, D, and J genes, is among the main enablers of the aforementioned diversity. In more detail, the Complementarity-determining region 3 (CDR3), which is formed at the junction of the recombined V, D, and J genes, is instrumental for the determination of the antigen binding ability of the T- or B-cell receptor.

*Immune repertoire profiling*, i.e., the study of TR and BcR repertoires, is a prerequisite for diagnosis, prognostication and monitoring of hematological disorders (e.g., various lymphoid malignancies [1, 2]) and it commonly includes the quantification of 1) the *diversity and expression of TR or BcR clonotypes*, i.e., the distinct clones of T or B receptor cells in a biological sample, and 2) the *V, D, J gene usage*, i.e., the frequency at which the various germline V, D, J genes have been rearranged to generate the TR or BcR clonotypes in the sample. The emergence of High-throughput sequencing (HTS) is a major enabler of complete and accurate immunogenetic repertoire profiling [3, 4].

The high demand of computational tools that facilitate the study of TR and BcR repertoires (*immune repertoire profiling software* from now on) is evidenced by the large number of available software (S/W) applications that undertake one or more steps to this direction. Downstream repertoire profile analysis usually starts with *receptor sequence annotation*, i.e., the spotting of the CDR3 within the receptor sequence and the identification of the germline genes of the V, D and J gene subgroups that have been recombined to form the receptor. IMGT/HighV-Quest [5, 6] and IgBLAST [7] offer online receptor sequence annotation services, while Decombinator [8], MiTCR [9] and MiXCR [10] are examples of command-line applications with the same mission. The next step in the analysis would be the *receptor repertoire quantification*, including tasks such as the extraction of the clonotype diversity and expression, the calculation of the V, D and J gene usage, etc. Advanced descriptive statistics and visualizations can then be easily extracted from quantified repertoires. Finally, *receptor repertoire comparison* functionalities are sometimes offered to search for similarities and/or differences between multiple repertoires.

In the context of immunogenetic profiling studies, there is no universally accepted way of defining TR and BcR clonotypes: Different clonotype definitions have been adopted by different studies, spanning from the complete receptor sequence to the CDR3 junction, which can be specified either at the nucleotide (NT) or the aminoacid (AA) level [11]. The *IMGT clonotype (AA)*, i.e., a unique tuple of the gene and alleles participating to a V(D)J rearrangement along with the CDR3 junction sequence (AA) [11], is probably the most prominent clonotype definition, having showcased its value in the comparison of both TR and BcR repertoires [12]. However, alternative, less detailed clonotype definitions have also been employed by a number of immune repertoire profiling applications [9, 10, 13].

The present study introduces a novel software pipeline for immune repertoire profiling of high-throughput TR and BcR sequencing data, called Immunogenetic Repertoire Profiler or **IRProfiler.** IRProfiler covers two of the aforementioned receptor repertoire analysis tasks, namely receptor repertoire quantification and comparison. The introduced pipeline adopts 5 alternative TR and BcR clonotype definitions to offer a list of core immune repertoire analysis functionalities. IRProfiler is implemented as a toolbox of the powerful web-based Galaxy platform [14, 15].

## Implementation

### Design considerations

In a crowded ecosystem of immune repertoire profiling software applications offering similar or identical functionalities, one option for a newly introduced application to prove its value is by trying to optimally satisfy user needs. The core immune repertoire profiling functionalities that are offered by IRProfiler are mostly shared with other pre-existing software applications. Therefore, we have adopted a user-centric approach in the design of the introduced pipeline so as to ensure that IRProfiler is **flexible, easy to use, easy to access,** while its **output is easily exploitable**.

The main design considerations that were taken into account while developing IRProfiler along with the decisions that were made to cater for these considerations are described below.

- **Flexibility.** In IRProfiler, we have attempted to ensure flexibility by offering a list of user options whenever possible (see for example the implemented data filtering criteria in Section Data filtering). Additionally, we have decided to support 5 alternative clonotype definitions (see Section Clonotype diversity and expression), i.e., an analysis parameter at the very core of IRProfiler’s repertoire quantification and comparison functionalities.
- **Ease of use**. Having to choose between a command-line and graphical user interface, we have opted for the latter, which is in general more appealing to novice users (e.g., immunogeneticists without strong technical background). On top of that, we have decided to implement the introduced pipeline as a toolbox of Galaxy, an established bioinformatics platform with a large community of users [16]. This allows IRProfiler to benefit from the straightforward, easy-to-use interface of the Galaxy platform.
- **Ease of access**. This consideration is associated with the distribution and possible installation of a software application. The installation and proper setup of native software applications can sometimes be challenging for technically inexperienced users (e.g., due to the presence of dependencies/requirements at operating system and/or application layer). Instead, a web-based approach, such as the one adopted for IRProfiler owing to its web-based hosting platform (i.e., Galaxy), means that all a user needs to use IRProfiler is internet access and an up-to-date web browser. The web-based approach is complemented by the 3 alternative distribution options that have been foreseen for IRProfiler (see Section Pipeline overview).
- **Output exploitability**. Same as in other bioinformatics subdomains, immunogeneticists and immunoinformaticians are most probably using several software applications to perform their end-to-end analyses (e.g., one application for receptor annotation, another for repertoire quantification, and a 3rd one for visualization of the quantification results). Moreover, they sometimes need to revisit certain steps of their analytical pipeline at future points. In all of these cases, it is important to have the final and intermediate results that are generated by a software application persistently stored in file types, formats and schemas that are easily exploitable by other applications. To this direction, each tool of IRProfiler has been designed to output all the outcomes of the conducted analysis in a small number of tab delimited files that pertain to straightforward – in the context of immune repertoire profiling – schemas (see Section Developed functionalities). Moreover, small summary files giving a quick overview of the conducted analysis are most of the times included in the list of outputs.

### Pipeline overview

Receptor sequence annotation, i.e., the first step of immune repertoire profiling analysis, is out of the scope of IRProfiler. Therefore, IRProfiler accepts as input annotated TR beta chain or BcR IG heavy chain HTS reads. IMGT/HighV-Quest [6] is the receptor sequence annotation tool of choice for IRProfiler. More specifically, among the 11 files that are outputted by IMGT/HighV-Quest, IRProfiler uses the *IMGT Summary Report*, i.e., a tabular file where each row corresponds to an annotated sequence read from the TR beta chain or BcR IG heavy chain DNA. The exact fields of the IMGT Summary report that are employed by the pipeline are listed in Table 1 and their semantics can be found in [17].

**Table 1:** Fields of the IMGT Summary Report that are employed by the introduced pipeline

Index	Field Name
1	AA JUNCTION
2	V-GENE and allele
3	V-REGION identity %
4	J-GENE and allele
5	D-GENE and allele
6	Functionality

Although only IMGT/HighV-Quest is explicitly supported, owing to the fact that the fields of Table 1 contain information that is commonly extracted during immune receptor annotation, any annotated high-throughput dataset that incorporates synonymous and semantically equivalent fields with those listed in Table 1 can also be used as input to the introduced pipeline. This fact significantly extends the application range of IRProfiler by allowing datasets annotated by other established immunogenetic annotation services (e.g., IgBLAST [7]) or custom annotation software to be analyzed, either as-is or after a proper schema transformation.

**Fig. 1:**
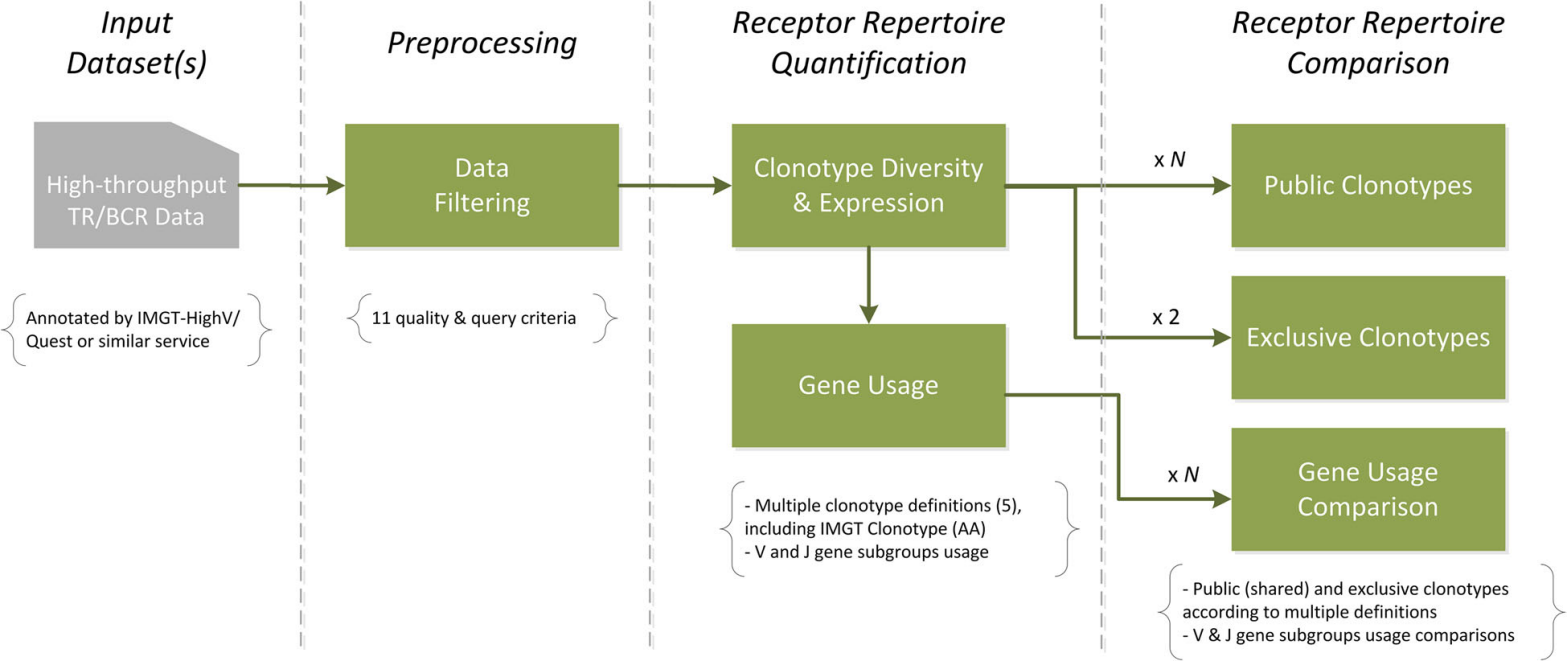
Conceptual design of IRProfiler

The conceptual design of IRProfiler is presented in Fig. 1. The functional building blocks (in green) of the pipeline correspond to the 6 tools of the IRProfiler toolbox and they are presented in the subsection that follows. The inputs and outputs of all tools are *tab delimited files*.

IRProfiler is distributed to the scientific community via three alternative options:

1. **Galaxy’s Main Tool Shed**. The developed tools have been published to the main Galaxy Tool Shed under a dedicated repository [18].
2. **Dedicated Galaxy installation**. IRProfiler has also been incorporated in a dedicated Galaxy installation that is deployed at [19]. A *Getting Started* guide is available on the homepage of the Galaxy installation.
3. **Galaxy Docker Image**. The dedicated Galaxy installation of the previous option which incorporates IRProfiler is freely available as a Docker image via the Docker Hub [20].

### Developed functionalities

This subsection describes the functionalities that are offered by IRProfiler and outlines the Galaxy tools that implement them. Conceptually, the *Clonotype diversity and expression* and the *Gene usage* functionalities are classified as receptor repertoire quantification tasks, while the *Public clonotypes*, *Exclusive clonotypes* and *Gene usage comparison* functionalities fall within the receptor repertoire comparison category. The *Data filtering* functionality can be considered as pre-processing task.

#### Data filtering

The mission of the data filtering functionality is twofold. First, to ensure that the annotated receptor reads that are going to be used in the quantification of the repertoire *satisfy certain immunogenetically-relevant quality criteria* (e.g., the CDR3 junction has the conserved anchors 104 and 118, the junction is in-frame, the V gene is functional and/or has been identified with a high certainty, the receptor read is productive, etc.). Filtering the annotated receptor reads on the basis of such criteria is of great significance, since the inherent limitations of both the wet-lab protocols and the HTS technologies result in a non-negligible portion of the outputted sequence reads being problematic. The second mission of the functionality is *querying the receptor dataset for reads with specific properties* (e.g., specific V or J gene participating in the V(D)J recombination, CDR3 length falling within a specific range or containing specific AA sequence, etc.). This use case allows the construction of on-demand subsets of the receptor read data to support specialized downstream repertoire-related analyses.

Eleven filtering criteria have been implemented. The Galaxy tool that implements this functionality receives as input 1 IMGT Summary Report file and, after applying the user-specified criteria, it outputs as single files 1) the filtered-in receptor reads, 2) the filtered-out receptor reads, along with the reason of their rejection, and 3) a short summary of the filtering outcome. At this stage, the allele information extracted by IMGT/HighV-QUEST is discarded (only the gene information remains).

Listing 1 Pseudocode abstracting the function of the data filtering tool^1^

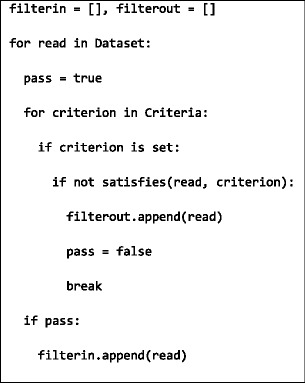



#### Clonotype diversity and expression

This functionality assigns each of the filtered-in receptor reads to a TR or BcR clonotype, so as to evaluate the clonotype diversity (i.e., the set of unique clonotypes) and clonotype expression (i.e., the frequency of receptor reads for each clonotype) of the investigated receptor repertoire.

Five alternative definitions of clonotypes are supported in this process, starting from the proven IMGT clonotype (AA) and gradually moving towards less detail. These are outlined in Table 2. According to each of the supported definitions, a clonotype corresponds to a *unique tuple of receptor properties.* For instance, the *V + J + CDR3 clonotype* corresponds to the triple (CDR3-AA, V-Gene, J-Gene), while the *CDR3 clonotype* is defined by a single property, i.e., the AA sequence of the CDR3 junction.

**Table 2:** List of clonotype definitions supported by IRProfiler

Index	Clonotype Name	Components	Comment
1	V + D + J + CDR3	(V-gene, D-gene, J-gene, CDR3-AA)	IMGT Clonotype (AA) with the allele information omitted
2	V + J + CDR3	(V-gene, J-gene, CDR3-AA)	No D-gene information; caters for D-gene assignment ambiguity
3	V + CDR3	(V-gene, CDR3-AA)	Specialized definition, focusing on V-gene
4	J + CDR3	(J-gene, CDR3-AA)	Specialized definition, focusing on J-gene
5	CDR3	(CDR3-AA)	The least detailed definition including only CDR3-AA

CDR3-AA denotes the animoacid translation of the CDR3 including the anchor animoacids (104 and 118)

From the algorithmic standpoint, after the desired clonotype definition is selected by the user, the filtered-in receptor reads are grouped by the unique tuple of properties/fields corresponding to the selected clonotype definition and the number of receptor reads in each group is calculated. The resulting groups are able to characterize the clonotype diversity, while the group counts determine the clonotype expression.

Listing 2 Pseudocode abstracting the function of the clonotype diversity and expression tool

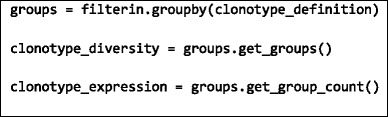



The tool that implements this functionality processes the filtered-in receptor reads produced by the data filtering tool to output 3 files: 1) the list of distinct clonotypes along with their frequency (absolute and relative) in decreasing order, 2) the top-10 clonotypes with the highest frequencies, and 3) a summary of the clonotype quantification outcome (i.e., the dominant clonotype and its frequency, the total number of clonotypes, the total number of expanding clonotypes, and the total number of singletons^2^). Although the information included in the last two files can be easily extracted from the contents of the first file, the former are provided as outputs of the tool to provide quick access to high-level summary information concerning the clonotype repertoire.

#### Gene usage

The objective of this functionality is to evaluate the usage of the germline genes participating in the V(D)J recombination process in an observed clonotype repertoire. More specifically, it calculates the *frequency at which each member of the V and J gene subgroup has been employed in a clonotype diversity repertoire.* The calculation of D gene usage is not supported by IRProfiler due to the high occurrence of ambiguities in D gene assignment (caused by additions or deletions of nucleotides at/from the ends of the recombining genes in conjunction with the short length of many D genes).

For each of the supported gene subgroups (V and J), IRProfiler iterates over the list of distinct clonotypes to calculate the absolute and relative (as percentage) frequency of each employed gene. Evidently, for the V (J) gene usage to be computed, the clonotype definition that has been used for producing the input clonotype diversity repertoire needs to include the V (J) gene. As a counterexample, the J gene usage cannot be computed if the V + CDR3 clonotype definition had been used for extracting the clonotypes in the previous step.

Listing 3 Pseudocode abstracting the function of the gene usage tool

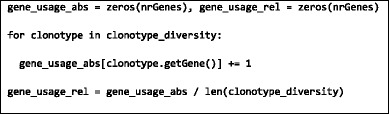



The tool that implements this functionality takes as input the 1st output of the clonotype diversity and expression tool (i.e., the complete list of distinct clonotypes). Following the same rationale as the previous tool, it generates 3 files: 1) the usage of all the employed V or J genes as absolute and relative frequencies, 2) the top-10 V or J genes with the highest frequencies, and 3) a summary of the gene usage computation outcome (i.e., the dominant gene in the subgroup and its frequency).

#### Public clonotypes

The mission of this functionality is *the discovery of shared clonotypes within multiple receptor repertoires*. Given 2 or more clonotype repertoires, the term *public* is used in this work to refer to a clonotype that is present in at least 2 repertoires. This functionality is supported for clonotype repertoires that have been extracted using the CDR3, V + CDR3 or J + CDR3 clonotype definition.

Assuming one of the 3 aforementioned clonotype definitions, IRProfiler outer joins the input individual clonotype diversity repertoires (2 or more) on the tuples that compose the assumed clonotype definition. The join operation preserves the relative frequency of the clonotypes in each of the individual clonotype repertoires. Then, for each joined clonotype, the number of individual repertoires it belongs to (repertoire count) is calculated; the joined clonotypes whose repertoire count is equal to 1 are filtered out (non-public).

Listing 4 Pseudocode abstracting the function of the public clonotypes tool

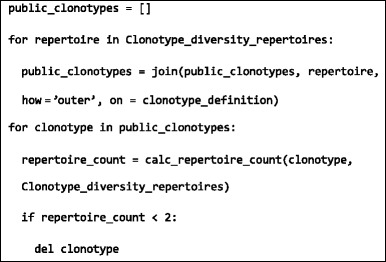



The public clonotypes tool processes a list of clonotype diversity repertoires (1st output of the clonotype diversity and expression tool) and it generates 1 output file containing the public clonotypes accompanied by their frequencies in each input repertoire and their repertoire count.

#### Exclusive clonotypes

This functionality *compares two input individual clonotype repertoires to detect the clonotypes that are exclusively found in the first repertoire* (i.e., they are absent from the second repertoire). Similarly to the previous functionality, only clonotype repertoires that have been extracted using the CDR3, V + CDR3 or J + CDR3 clonotype definitions can be processed by the present functionality.

Assuming one of the 3 aforementioned clonotype definitions, the detection of exclusive clonotypes is implemented as a left join between the two input individual clonotype diversity repertoires on the tuples that compose the assumed clonotype definition followed by the removal of the joined clonotypes with non-zero frequency in the second repertoire.

Listing 5 Pseudocode abstracting the function of the exclusive clonotypes tool

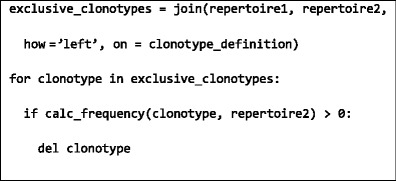



The present tool processes 2 input clonotype diversity repertoires (1st output of the clonotype diversity and expression tool) and it generates 1 output file containing the exclusive clonotypes of the 1st repertoire.

#### Gene usage comparison

Similarly to the way the clonotype repertoires are compared as part of the public and exclusive clonotypes functionalities, *multiple V or J gene repertoires can be compared with respect to the gene usages*. This is the objective of the present functionality. More specifically, given 2 or more V or J gene repertoires, the discussed functionality places side by side the usage of the genes of the subgroup in each repertoire and it also calculates the mean gene usage across all repertoires.

An outer join of the input gene usage repertoires (2 or more) on the V or J gene followed by the calculation of the mean usage of each joined gene across all input repertoires implements the discussed functionality.

Listing 6 Pseudocode abstracting the function of the gene usage comparison tool

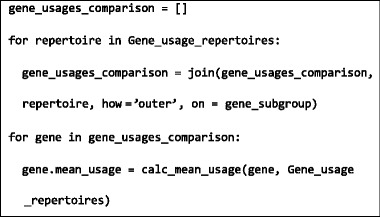



The gene usage comparison tool processes a list of gene usage repertoires (1st output of the gene usage tool) and it generates 1 output file containing for all the employed genes their usages in each input repertoire and their mean usage across all input repertoires.

## Results and discussion

From the presentation of the IRProfiler functionality in the previous section, it becomes clear that the extraction of clonotype diversity and expression lies at the core of the introduced pipeline. The adoption of multiple clonotype definitions with different levels of detail adds a level of analysis flexibility to IRProfiler, which is not given in immune repertoire profiling software. Accepting the IMGT clonotype (AA) as the prevalent choice for clonotype definition, there are several cases where one of the alternatives might be more appropriate. For instance, for an immune repertoire with high percentage of ambiguous D gene assignments it might be preferable to use the V + J + CDR3 clonotype definition instead. Other examples originate from the particular study target of an attempted analysis: If one wishes to compare two distinct CDR3 repertoires, it is reasonable to start by selecting the CDR3 clonotype definition in the clonotype diversity and expression tool.

The integration of IRProfiler in Galaxy allows the introduced pipeline to benefit from the *usability* of the hosting platform. The tools of IRProfiler can be manually invoked sequentially in a user friendly manner. However, workflows combining explicitly ordered invocations of several tools with specific parameters can also be configured by the user.

The description of the developed tools in the previous section has shown that both the receptor repertoire quantification and comparison functionalities are implemented via unambiguous data manipulation techniques. Each developed tool was unit tested with the help of reference input and output data. More specifically, for this purpose pairs of small-scale input datasets and expected output datasets (manually generated) were compiled for each tool. A part of the employed reference input and output datasets has been made available to the readers of this article (see Section Availability of data and material).

### Scalability

In order to assess the *scalability* of IRProfiler, the developed tools were *stress-tested* with respect to the **execution time** and **peak memory usage** (i.e., the maximum RAM memory that is instantaneously needed during the execution) on a wide range of – realistic – input dataset sizes via a series of in silico experiments. The specifications of the hardware and software employed in the experiments is listed in Table 3. In addition to the experimentally determined actual execution times, their theoretical upper bounds for each tool were also estimated.

**Table 3:** Specifications of the hardware and software setup for the scalability evaluation experiments

Processor	Intel(R) Core(TM) i7–4790 CPU @ 3.60GHz, × 64
RAM Memory	16 GB RAM DIMM DDR3 Synchronous 1600 MHz
Storage	INTEL SSD SC2BW18, SATA 3.0 6Gbs
OS	Ubuntu 16.04.1 LTS
Python & Libraries	CPython 3.4.5 with Pandas 0.19.1

For the scalability analysis, the developed tools were classified into two categories: *single input tools* (data filtering, clonotype diversity and expression, gene usage) and* multiple input tools* (public clonotypes, exclusive clonotypes, gene usage comparison). The experiments for the tools of the 2nd category were conducted with exactly 2 input datasets.

#### Execution time

As a theoretical exercise, the upper bound of the execution time was theoretically estimated for each tool based on the underlying algorithm (see Section Developed functionalities). The resulting estimations are provided in the 3rd column of Table 4 by means of the *O*(·) notation, indicating the linear and quadratic relation of the execution time with the size of the input dataset(s) for the single and multiple input tools, respectively.

**Table 4:** Results of theoretical and experimental execution time estimation (extracted independently) for the developed tools

Index	Tool	*O*(·)	*R* ^2^
1	Data filtering	*O*(n)	0.999808
2	Clonotype diversity and expression	*O*(n)	[0.999508, 0.999717]
3	Gene usage	*O*(n)	[0.976990, 0.981139]
4	Public clonotypes	*O*(m·n^2^)	[0.976313, 0.996352]
5	Exclusive clonotypes	*O*(n^2^)	[0.937594, 0.958152]
6	Gene usage comparison	*O*(m·n^2^)	[0.834540, 0.864164]

For the theoretical estimation (3rd column), *n* is the number of input receptor reads or clonotypes and *m* is the number of input repertoire datasets. For the experimental estimation (4th column), exactly 2 input datasets have been assumed for the 4th–6th tools. The 4th column includes the coefficient of determination values (*R*^2^) assuming a first (1st-3rd tool) and second (4th–6th tool) order polynomial model of the execution time; whenever multiple alternative clonotype definitions or gene subgroups are supported by a tool, ranges of values are reported

Independently of the theoretical estimations, the actual values of the execution time of each tool on gradually increasing artificial input datasets were recorded. Whenever multiple clonotype definitions or gene subgroups were supported by a tool separate execution times were recorded for each available option. The recorded execution times were then fitted to a first or second order polynomial model for the single input and multiple input tools, respectively.

The coefficient of determination (*R*^2^), i.e., the percentage of the response variable variation that is explained by a selected model [21], was employed to assess the validity of the linear or quadratic relation hypothesis (see last column of Table 4). For the most part, the experimental results back up the findings of the theoretical estimation, which can only be questioned for the case of the gene usage comparison tool (*R*^2^ value around 0.85).

#### Peak memory usage

**Fig. 2:**
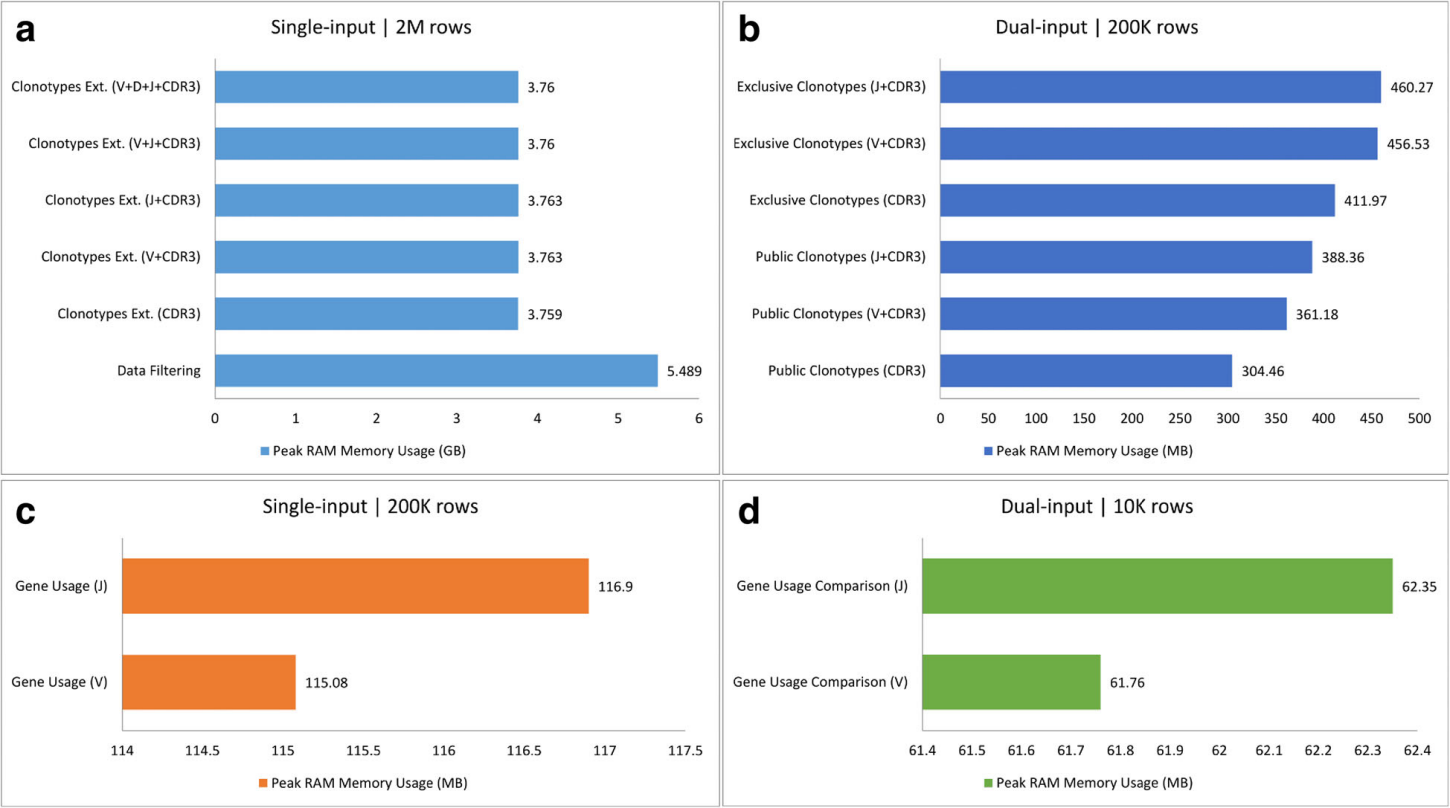
Bar charts of peak RAM memory usage for various groups of tools – tools are grouped on the basis of number of inputs and their size (in number of rows; M = 10^6^, K = 10^3^)

With respect to the peak memory usage, the value of the metric for reasonably large artificial input dataset(s) was recorded for each tool. This essentially corresponds to the most memory-demanding task each tool will have to carry out in a realistic usage setting. The measured peak memory usage is visualized in Fig. 2, where the tools are grouped on the basis of 1) the number of inputs, and 2) the size of the input datasets. Of note, even the memory requirement that is reported by the most memory-consuming tool (data filtering tool; almost 5.5 GB of RAM) is manageable for a modern data processing workstation or server.

### Comparison with existing software

Since IRProfiler targets exclusively receptor repertoire quantification and comparison, it should be compared with software applications that deal with one or both of the aforementioned immune repertoire profiling tasks. A thorough review of the literature has helped us identify the following list of software applications falling within the former description: **IMGT/HighV-QUEST** (Statistics tab) [11], **IGGalaxy** [22], **tcR** [23], **IMonitor** [24], **IMSEQ** [25], **IMEX** [26], and **Vidjil** [27]. Table 5 provides a structured way of comparing these applications with IRProfiler in terms of functionality and other software properties.

**Table 5:** Comparison of IRProfiler with existing software with respect to functionality and other S/W properties

		IMGT	IGGalaxy	tcR	IMonitor	IMSEQ	IMEX	Vidjil	IRProfiler
**Properties**	**S/W Interface**	Graphical	Graphical	Command-line	Command-line	Command-line	Graphical	Graphical	Graphical
	**S/W Type**	Web-based (Asynchronous)	Web based (Galaxy)	Native (R package)	Native (Shell script)	Native (Shell script)	Native (C# executable)	Web-based	Web based (Galaxy)
	**TR/BcR**	+/+	−/+	+/−	+/+	+/+	+/+	+/+	+/+
	**Output Files**	Hundreds of files (HTML, PDF, PNG)	Tab delimited text and HTML files	R dataframes (can be saved to text)	Several PDF files	1 Tabular text file (optionally PDF graph)	Tabular text and image files	Various files (HTML, JSON, CSV, PDF, FASTA)	Tabular text files (2 or 3 per tool)
	**Clonotype Definition(s)**	IMGT Clonotype (AA)	V + CDR3 (AA); V + CDR3 (NT); V + J + CDR3 (NT); V + D + J + CDR3 (NT)	IMGT Clonotype (AA) – works also with “relaxed” definitions	CDR3 (AA); CDR3 (NT)	V + J + CDR3 (AA)	CDR3 (AA); CDR3 (NT); V + D + J (incl. allele); whole read (NT | AA)	V(D)J junction (NT) – also supports 3rd S/W definitions	5 definitions (see Table 2)
**Functionalities**	**Data Filtering**	Conserved anchors; V/J functional; ORF	Productive reads	User-specified filtering supported	Pseudogenes; out-of-frame; stop codons; etc.	Conserved anchors; out-of-frame; stop-codons	IMGT “no result”	Conserved anchors	11 read quality criteria
	**Clonotype Extraction**	Clonotype diversity and expression	Clonotype diversity	Clonotype diversity and expression	Clonotype diversity and expression	Clonotype diversity and expression	Clonotype diversity and expression	Clonotype diversity and expression	Clonotype diversity and expression
	**Gene Usage Calculation**	V, D and J gene subgroups	V, D and J gene subgroups	V and J gene subgroups	V and J gene subgroups	N/A	V, D and J gene subgroups	N/A	V and J gene subgroups
	**Clonotypes Comparison**	Public clonotypes and number of exclusive ones	N/A	Public clonotypes	N/A	Top 10 public clonotypes	Top N (user defined) public clonotypes	Public clonotypes	Public and exclusive clonotypes
	**Gene Usage Comparison**	V, D and J gene subgroups	V, D and J gene subgroups	Entire V and J gene usage repertoire comparison	N/A	N/A	N/A	N/A	V and J gene subgroups
	**Others**	Various diversity and expression histograms (e.g., per CDR3 length), etc.	V-D, V-J and D-J gene combination heatmaps	Advanced statistics for diversity and gene usage, visualizations, etc.	Receptor annotation, error correction, visualizations	Clonotype clustering (for ambiguity resolution)	V-J gene combination heatmaps, primer efficiency analysis	Receptor annotation, Interactive visualization	N/A

**Fig. 3:**
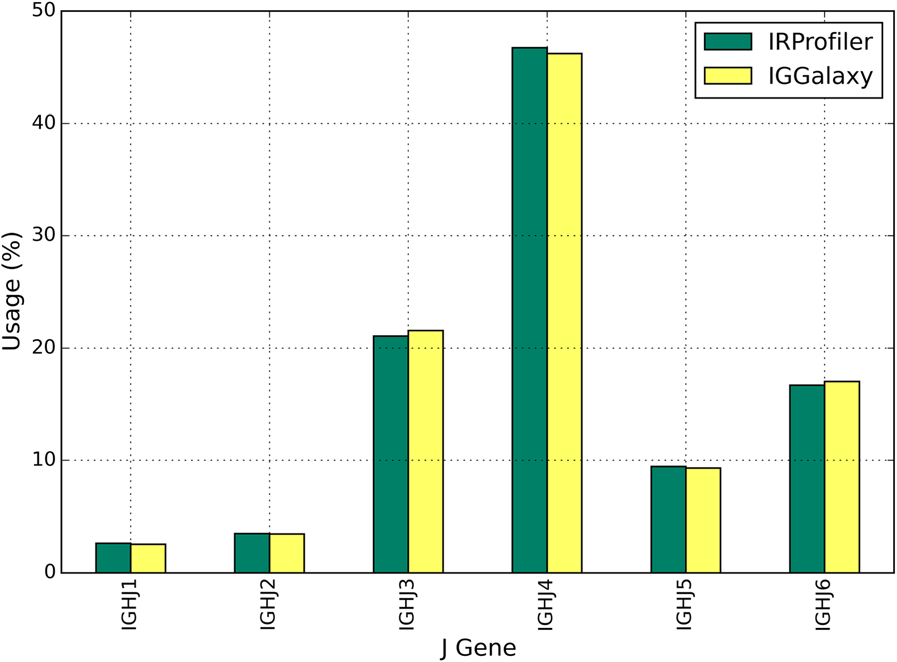
Bar charts of the J gene usages that are calculated by IRProfiler and IGGalaxy for a public BcR dataset

The study of Table 5 reveals that, in an ecosystem of heavily overlapping immune repertoire profiling applications, most of the functionalities of IRProfiler are also offered by pre-existing software for a subset of the clonotype definitions that are supported by this work. Indeed, the utilization of the aforementioned software for analyzing public TR or BcR datasets has verified that – unsurprisingly, given the type of the analysis – the obtained results from shared functionalities and clonotype definitions (clonotype diversity and expression, gene usage, etc.) are very similar or identical with those produced by IRProfiler. As an example, the J gene usages that are calculated for a public BcR dataset [28] by IRProfiler and IGGalaxy are visualized as bar charts in Fig. 3. Another example comes from the comparison of IRProfiler with tcR using a public TR dataset [29]. For this comparison, we randomly extracted from the public datasets two subsets of 300 K reads each and fed them to the two applications. In this case as well, the V gene usages calculated by the two applications are almost identical; moreover, the two applications reported practically the same number of public V + CDR3 clonotypes (this functionality is called *repertoire overlap* in tcR): 11,430 by tcR versus 11,436 by IRProfiler.

A comparison of IRProfiler with the aforementioned software applications regarding the execution speed is difficult to implement, due to the diversity of their deployment and execution environments (including native, web-based and virtualized applications). Although most of these software applications are quite fast, a similar argument can be made for IRProfiler on the basis of its good scalability performance (see Section Scalability). In any case, potential differences in execution times between fast and scalable immune repertoire profiling applications are not expected to have an impact on the user experience, given the intended usage of the software (i.e., exploratory and research oriented high-throughput data analysis software). Concerning a comparison of the ease-of-use, quantifiable conclusions cannot be drawn either, for the same reasons as above. However, it is worth mentioning once more that the ease-of-use objective was been taken into account in the design of IRProfiler (see Section Design considerations).

### Case studies

IRProfiler has been extensively used by the Health Translational Research group of the Institute of Applied Biosciences of the Centre for Research & Technology Hellas through an in-house Galaxy installation for the conduction of a number of case studies. So far, several publications have exploited IRProfiler mainly to investigate the restrictions in the repertoire of TR in various hematological disorders, attesting to the value of the present work for immunogenetics researchers. More specifically, the repertoire of TR in Chronic idiopathic neutropenia (CIN) has been studied in [30] assuming the V + CDR3 clonotype definition (employed tools: data filtering, clonotype diversity and expression, gene usage, public clonotypes). For the case of Chronic lymphocytic leukemia (CLL), the TR repertoire has been the study subject in [31] and – its extended version – [32]. These works also adopted the V + CDR3 clonotype definition (employed tools: data filtering, clonotype diversity and expression, gene usage, public clonotypes, exclusive clonotypes). Finally, the developed toolbox has been utilized in [33] to study the TR repertoire in Paroxysmal nocturnal hemoglobulinuria (PNH); in the last study, the J + CDR3 clonotype definition was adopted (employed tools: all 6 developed tools).

Apart from the aforementioned studies, an implementation of IRProfiler for the Apache Spark [34] cluster-computing framework has been integrated in the big data analytics platform that is being developed by AEGLE [35], an ongoing EC-funded collaborative research programme.

## Conclusions

IRProfiler is a new entry in the ecosystem of immune repertoire profiling applications providing core quantification and comparison functionalities on annotated TR beta chain or BcR IG heavy chain HTS data. The **support of 5 clonotype definitions of different levels of detail**, including the proven IMGT clonotype (AA), along with several data filtering criteria offer the users of IRProfiler a considerable flexibility in immune repertoire profiling analysis.

Although most of the offered functionalities of IRProfiler can be found in pre-existing software applications (at least for some of the supported clonotype definitions), the introduced pipeline brings added-value for immunogeneticists and immunoinformaticians based on a **particular combination of design properties**: The web-based distribution of IRProfiler (complemented by other attractive distribution options), its graphical user interface, the easily exploitable tab delimited files outputted in every step of the analysis, and, of course, the aforementioned flexibility in the analysis stem from the user-centric design of IRProfiler.

The selection of Galaxy as the hosting platform of IRProfiler ensures the usability and modularity of IRProfiler and provides a powerful means for its distribution (i.e., Galaxy Tool Shed). From a technical standpoint, IRProfiler seems to scale well (checked both theoretically and experimentally) with respect to the size of its input datasets, a feature that is particularly relevant in HTS data analysis settings. The introduced pipeline has already been employed by a number of publications for TR repertoire profiling in various hematological disorders.

## Availability and requirements

**Project Name:** IRProfiler.

**Project home page:** http://irprofiler.med.auth.gr:8080/

**Operating system(s):** Platform independent.

**Programming language:** Python.

**Other requirements:** Python 2.7 or higher, Pandas 0.19 or higher.

**License:** GNU GPL.

**Any restrictions to use by non-academics:** None.

## Abbreviations

AA: Aminoacid
BcR: B-cell receptor
CDR3: Complementarity-determining region 3
CIN: Chronic idiopathic neutropenia
CLL: Chronic lymphocytic leukemia
HTS: High-throughput sequencing
IG: Immunoglobulin
IMGT: international ImMunoGeneTics information system
NCBI: National Center for Biotechnology Information
NGS: Next generation sequencing
NT: Nucleotide
PNH: Paroxysmal Nocturnal Hemoglobulinuria
S/W: Software
TR: T-cell receptor

## Footnotes

1 A Python-inspired syntax has been used for all snippets of pseudocode in the manuscript.

2 A clonotype is characterized as expanding if it is represented in a dataset by at least 2 reads; otherwise, it is considered a singleton.
